# Implementation and Evaluation of Postpartum Midwifery Care at Home at a Federally Qualified Health Center in Washington, DC

**DOI:** 10.1177/24731242251382349

**Published:** 2025-10-01

**Authors:** Katie DePalma, Ebony Marcelle, Emma Clark, Teresa O’Brien, Sanjana Chimata, Erica Eliason, Bayyinah Muhammad, Sola Stamm, Nicole Vernot-Jonas, Pawel Olowski, Dawn Sherman, Christina X. Marea

**Affiliations:** ^1^ School of Nursing, Georgetown University, Washington, District of Columbia, USA.; ^2^ Community of Hope, Washington, District of Columbia, USA.; ^3^ Center for State Health Policy, Rutgers University, New Brunswick, New Jersey, USA.; ^4^ Department of Global Health, School of Health, Georgetown University, Washington, District of Columbia, USA.

**Keywords:** postpartum, perinatal, health inequities, home care, midwifery, FQHC

## Abstract

**Background:**

The postpartum period is a critical time to support birthing people and infants, address and mitigate perinatal health inequities, and promote joy and connection. Yet many birthing people do not perceive value in postpartum visits, particularly when measured against the competing demands and barriers to care faced by those experiencing poverty and other systemic oppression. These inequities are particularly acute for Black birthing people insured by Medicaid due to structural and interpersonal racism, as well as economic disenfranchisement. We implemented an opt-in postpartum care at home program led by midwives at a federally qualified health center to increase the perceived value of postpartum care and increase patient engagement.

**Methods:**

We evaluated implementation using the reach, effectiveness, adoption, implementation, and maintenance framework. We assessed reach and effectiveness using electronic health record data for 2022 (pre-implementation) and 2023 (implementation).

**Results:**

Among 473 birthing people eligible during the evaluation period, 118 (28%) had a postpartum home care visit. Postpartum visit attendance rates increased from 78% in 2022 to 84% in 2023. In-home postpartum care increased family involvement, enabled longer and more comprehensive visits, and improved provider satisfaction.

**Conclusions:**

Postpartum care at home can reduce barriers to care, increase visit attendance, and promote joy. In-home postpartum care with a midwife has the potential to mitigate health inequities experienced by Black birthing people by fostering trust and shifting the power dynamic in the patient–provider relationship. Community-based care must be designed and implemented with antiracist and emancipatory approaches.

## Introduction

Community-based midwife-led postpartum care is an intervention that can address deep inequities and worsening maternal mortality for Black birthing people while promoting personalized patient- and family-centered respectful care.^1–3^ Health equity, as defined by epidemiologist and physician Dr. Camara Jones, is the assurance of the conditions for optimal health for all people.^4^ We see health equity in the postpartum period as an action and an ongoing choice to prioritize justice and remove barriers. Black birthing people in the United States understand the impact of racism and structural inequities on their postpartum experiences, and have a clear vision for how comprehensive and joy-centered postpartum care can be accomplished.^5^ Postpartum care must become more accessible and valuable for those facing structural barriers to access (e.g., transportation, childcare, racism).^6^ Postpartum care at home can realign the power dynamics of a medical system that has historically disregarded or outright harmed Black birthing people.^7,8^

The postpartum period is characterized by immense transitions in physical, mental, social, and material well-being, transitions whose risks can be exacerbated by exposures to racism, poverty, housing instability, and lack of worker protections.^9^ Early comprehensive postpartum care (from 2 to 40 days) is essential to prevent complications such as postpartum hemorrhage and infection, and to identify risks to short- and long-term physical and mental health outcomes.^10^ Postpartum care can significantly decrease the incidence of postpartum depression and anxiety, enhance parent–infant bonding, and promote healthy parenting practices.^3,11,12^

The U.S. maternal mortality due to postpartum complications including severe hemorrhage, hypertensive disorders, and mental health (suicide and overdose)^13^ have risen in recent years, and while overall mortality has declined, it has worsened for Black birthing people.^14^ Gaps in postpartum care are missed opportunities for promoting maternal and infant well-being, often exacerbating existing health inequities.^15^ Black and Latinx individuals are disproportionately affected, with higher rates of postpartum complications and less access to midwifery care and postpartum care.^16,17^ Evidence continues to accumulate on the importance of addressing systemic barriers to care, suggesting that integrated models such as midwifery continuity of care with postpartum home visits can enhance access and reduce disparities.^8,18^

Postpartum care delivered within a continuity of care model, wherein a midwife or group of midwives maintains a consistent relationship with the birthing person throughout the perinatal period, is associated with enhanced patient satisfaction and lower rates of postpartum complications.^19,20^ Care delivered in a familiar environment can foster patient comfort and security.^21^ In these visits, a provider travels to the patient to provide essential postpartum care, assess the birthing person and baby, offer lactation support, and address concerns or complications that have arisen. This holistic approach, which addresses physical health, emotional well-being, and social support networks, is crucial during the transitional postpartum period.^22–24^

Most initiatives in the United States focus on the necessary, but inappropriately low, standard of surviving pregnancy and the postpartum year.^25^ For this program, we are centering thriving and joy. In our intervention design and implementation, we were cognizant of, and aimed to address, the root causes of health inequities for Black birthing people—less access to care, being served by poor quality institutions, and worse treatment within those institutions.^16,26^ This shift centers survival and health along with thriving Black joy and wellness, as defined by Black birthing people themselves.

## Methods and Materials

### Study setting

**FIG. 1.**
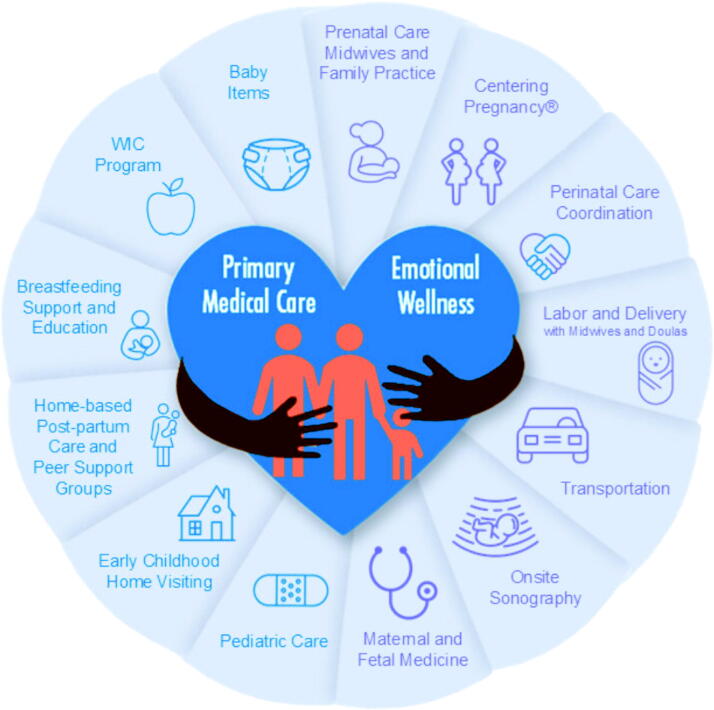
Maternal and Child Health Services Model at Community of Hope.

Community of Hope (COH) is an innovative federally qualified health center (FQHC) in Washington DC that surrounds patients and families with health and social resources, emphasizing nurturing community relationships. COH offers comprehensive services including midwifery-led continuity of care for pregnant clients, group prenatal and well child care, primary care, perinatal and sexual health care coordination, integrated emotional wellness (IEW) including therapy, supplemental nutrition for women, infants, and children (WIC), transportation, housing support, and lactation and doula support (see Fig. 1). COH midwives attend births at a tertiary care hospital and a freestanding birth center (one of three accredited FQHC-operated freestanding birth centers in the United States).^27^ In 2023, COH completed an average of 306 prenatal visits, 25 births, 86 postpartum visits, and 81 newborn visits per month.

### Theoretical framework

Our postpartum care initiative was guided by the 5D cycle for health equity, which joins appreciative inquiry, Black feminism, and radical imagination.^28^

### Stakeholder engagement

We conducted eight focus groups between February and April 2022—four with Black birthing people who had given birth in the past 2 years (*n* = 22), and four with health care providers and staff (*n* = 20) working at COH. One of the themes emphasized how difficult and overwhelming it can be to attend in-person care in the early postpartum period. Participants noted challenges with childcare for older kids (particularly in the absence of a social support network), navigating public transportation, extreme weather (heat, cold, fire), and lactation support. They also expressed a deep desire for racially and culturally congruent postpartum care that was attentive to the ways Black families are policed in the health care system.^29^ Following data analysis and member checking with patients and COH staff, we selected in-home postpartum midwifery care to minimize barriers to access, promote patient comfort and rest, and enable families and support people to be involved in care. Our clients were clear that in-home care must be optional, with no client feeling pressure to accept a health worker in their home.

### Conceptual model

We used the reach, effectiveness, adoption, implementation, maintenance framework to evaluate the implementation of the in-home postpartum care program (see Table 1).^30^

**Table 1.** RE-AIM Evaluation of In-Home Postpartum Midwifery Care

Evaluation question	Description	Data source
Reach		
How many clients participated?	131 postpartum patients (*N* = 473 eligible) 132 newborns (*N* = 478 eligible)	EHR data reports
How did those who participated differ from those who did not?	Patients who elected in-home care tended to align with four profiles: 1. Chose COH for the midwifery-led continuity of care model 2. Engaged with COH services (i.e., group care, integrated emotional wellness, and perinatal care coordination) 3. Had birth-related complications or unexpected social circumstances 4. Experienced structural and social barriers that were addressed by in-home care Patients with low engagement tended to align with two profiles: 1. Preferred language not English 2. Those with unstable housing	Implementation meeting minutes
Effectiveness		
What were the outcomes of implementing in-home postpartum midwifery care?	1. Increased postpartum visit attendance 2. High patient satisfaction and acceptability. 3. Reduced barriers to postpartum care	EHR data reports and implementation meeting minutes
Adoption		
What were organizational factors of engagement?	1. Engaged COH teams: leadership, midwifery, MCH, IEW, and QI 2. Increased clinic availability for midwifery care 3. Midwives and PCCs optimized workflows and communicated patient priorities and facilitated meeting needs 4. IEW providers virtually available “warm hand-offs” for mental health 5. Increased job satisfaction for home visiting midwives with longer visit times allowing more relationship-building, attunement, and comprehensive whole-person family-centered care	Implementation Meeting Minutes
Implementation		
What were barriers to implementation and what adaptations were made?	1. Location-reside in DC and MD, within a 30-min drive (MD) 2. LARCs and vaccines unavailable at home 3. Staff-prioritize daily clinic coverage, 24/7 coverage of two birth locations, and sick/vacation/parental leaves 4. Scheduling-2-h visit windows instead of specific appointment times, consider route planning	Bi-weekly implementation meeting minutes
Maintenance		
What happened to the program after completion of the study?	Incorporation of in-home care into midwifery practice as a standard offering with availability 1–2 days per week.	

COH, Community of Hope; EHR, electronic health record; LARCs, long-acting reversible contraceptives; QI, quality improvement; RE-AIM, reach, effectiveness, adoption, implementation, and maintenance; IEW, integrated emotional wellness.

### Program theory

**FIG. 2.**
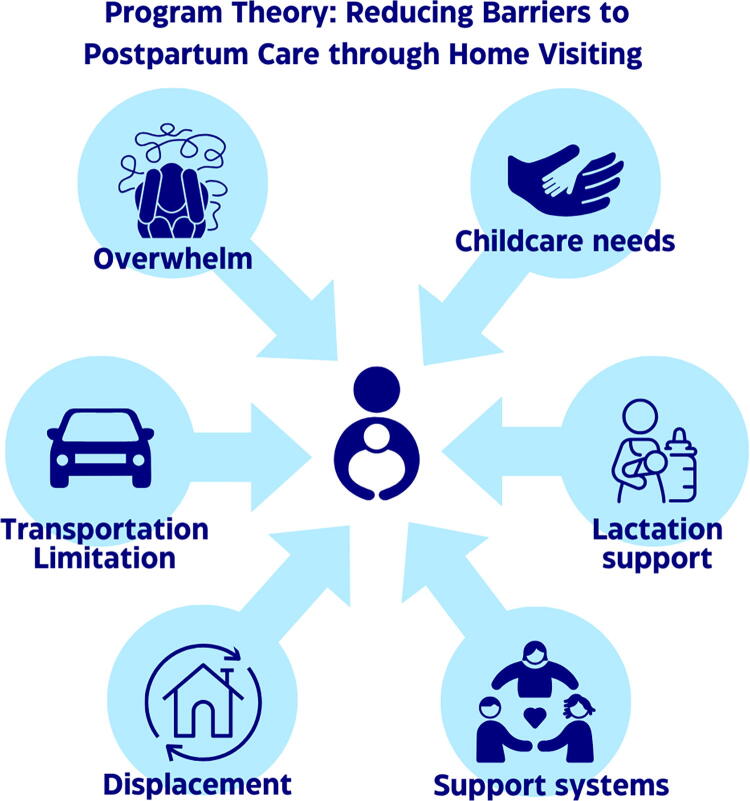
Program Theory: Reducing Barriers to Postpartum Care through Home Visiting.

Our program theory conceptualizes in-home postpartum care as a care modality to address barriers including transportation, overwhelm/competing demands, childcare, lactation support, and the economic and structural displacement of Black people and their support systems in DC (see Fig. 2). A provider traveling to the patient’s home has the potential to remove the need for transportation and childcare, ease logistical overwhelm that comes with an in-clinic visit, provide longer visits with additional education and lactation support, and allow patients to have support people present while prioritizing their own health care. With fewer barriers, we expected to see an increase in care engagement and patient satisfaction.

### Implementation team

The implementation team included a research lead who is also a per diem midwife at COH, and COH staff and providers including representatives from midwifery, maternal child health services (which includes care coordination, WIC, and other social supports), emotional wellness, quality improvement, scheduling and administration, and executive leadership. Input and feedback from all teams allowed iterative developments and streamlined integration into the COH care continuum. Beginning in April 2022, our team held biweekly collaborative meetings for planning and initiation of the in-home postpartum care offering. The three midwives who provided the home visits are monolingual (English).

### Description of the intervention

We designed our postpartum midwifery care at home program to center the needs, preferences, and experiences of our patients and the staff and providers at COH, integrating findings from formative qualitative work we conducted with Black birthing people.^5^ Patients were eligible for postpartum midwifery care at home if they had one perinatal encounter with a COH provider (prenatal visit, intrapartum care, and/or birth), and lived in DC or Maryland (∼30 min drive time limit). Upon giving birth, COH clients received a phone call from the perinatal practice manager to schedule their postpartum and newborn visits. Eligible clients were offered the option of postpartum care in the clinic or at home. Postpartum care at home was available from 2 to 84 days for the birthing person, and from 2 to 28 days for the newborn, and there was no limit set for the number of home visits an individual could receive during those timeframes. The timeframe for postpartum visits was 84 days (or 12 weeks), which was aligned with the HEDIS definition of the postpartum period for quality measures. The timeframe for newborn visits was 28 days because CNM scope of practice includes care of the newborn up to 28 days old. The only routine services not available at home (vs. clinic) were vaccines and long-acting reversible contraceptives (LARCs). See Table 2 for details on postpartum care at COH. Home visits were billed to Medicaid and private insurance using evaluation and management codes based on complexity, face-to-face time, and new versus established status as a patient. The most common current procedural terminology codes used were 99350 (established patients) and 99344 (newborn initial visits).

**Table 2.** Postpartum care at Community of Hope: Clinic vs. Home

Components of visit		
	Postpartum care-birthing person	
Visit component	Clinic	Home
Vital signs	X	X
History and physical assessment	X	X
Patient education and anticipatory guidance	X	X
Follow-up planning	X	X
Depression screening	X	X
Prescriptions, referrals, pap smear, and labs/blood work	X	X
Warm hand off to perinatal care coordinators, emotional wellness team,	In-person	Virtual (Video or phone)
Interpretation services	X	X
Lactation support	X	Expanded
Vaccines	X	—
Newborn and personal care supplies (i.e., diapers, breast pumps, layette sets, etc.), as available	X	Delivery of home supplies
Contraception	All methods including LARC insertion	Pills/patch/ring and injectable methods only

	Postpartum care-newborn	
Vital signs	X	X
History and physical assessment	X	X
Patient education and anticipatory guidance	X	X
Follow-up planning	X	X
Prescriptions, referrals, and labs/blood work	X	X
Newborn metabolic screening	X	X
Vaccines	X	—

	**Postpartum visit schedule-home vs. clinic**		
	**3–5 Days**	**2 Weeks**	**5–6 Weeks**
	**Birthing person**		
Home	+	S	S
Clinic	−	S	S
	**Infant**		
Home	S	S	^*^
Clinic	S	S	^*^

^*^ Current CNM scope of practice for care of the newborn is up to 28 days old.

COH, Community of Hope; S, standard visit schedule; +, expanded postpartum care.

### Study team positionality and reflexivity

The first author is a queer, partnered, nonbinary, able-bodied, neurodiverse white Irish-American nongestational parent. The cosenior authors include a Black American, cisgender, heterosexual, thought leader, activist, midwife scientist/director, wife, and mother; and a bilingual (English/Spanish) Latina, cisgender, heterosexual, neurodiverse parent who has given birth twice. We have all received postpartum home visits as a birthing person or partner. Our identities and experiences inform our belief in the importance of community and social support for postpartum people. We affirm the interconnectedness of our own liberation with that of all structurally excluded communities, notably Black femmes who have long stood at the front of liberation movements.

### Study design and sample

We conducted a retrospective review of deidentified electronic health record (EHR) data for 2022–2023 and structured implementation notes to evaluate the implementation and outcomes of the postpartum midwifery care at home initiative. The patient population included pregnant people (and their newborns) who received prenatal care at COH with estimated due dates (EDDs) between January 1, 2022, and December 31, 2023, and had a live or stillbirth.

### Ethics

This study was reviewed by the Georgetown University Medical Center IRB and was designated exempt nonhuman subjects research IRB study #STUDY00007090.

### Analysis

COH staff extracted EHR data on HIPAA-compliant devices from the EClinicalWorks health record system using Relevant, a web-based data management system. We performed data analysis using structured query language code to confirm reach and impact via visit attendance for postpartum people (2–84 days) and newborns (2–28 days). We used structured implementation meeting notes to examine adoption, implementation, and maintenance.

## Results

### Reach

There were 473 birthing people with EDDs in 2023 who were eligible for postpartum care at home. Eligible clients were predominantly Black (73%) adults 26–35 (50%) living below the poverty line (63%), and insured via Medicaid (65%). People with private insurance, non-Latino, English-speaking, and nulliparous clients were seen at home at higher rates than in the clinic. Among eligible birthing people, 118 (33%) elected to have at least one postpartum visit at home, and 132 newborns (including twins) had at least one newborn visit at home. See Table 3 for client demographic information.

**Table 3.** Client characteristics (birthing people with EDDs in 2023 who had live births and stillbirths)

Characteristic	All birthing people (*N* = 473)	Postpartum-home *n* = 118	Postpartum-clinic *n* = 355
Age			
≤18	8 (1.7%)	2 (1.7%)	6 (1.7%)
19–26 years old	150 (31.7%)	31 (26.3%)	119 (33.5%)
27–35 years old	239 (50.5%)	64 (54.2%)	175 (49.3%)
>35 years old	76 (16.1%)	21 (17.8%)	55 (15.5%)
Race			
American Indian/Alaska Native	3 (0.6%)	0 (0.0%)	3 (0.8%)
Asian	3 (0.6%)	1 (0.8%)	2 (0.6%)
Black/African American	347 (73.4%)	95 (80.5%)	252 (71.0%)
More than one race	3 (0.6%)	2 (1.7%)	1 (0.3%)
Other Pacific Islander	14 (3.0%)	0 (0.0%)	14 (3.9%)
Declined to report	68 (14.4%)	8 (6.8%)	60 (16.9%)
White/Caucasian	35 (7.4%)	12 (10.2%)	23 (6.5%)
Ethnicity			
Hispanic or Latino/a/e/x	83 (17.5%)	8 (6.8%)	75 (21.1%)
Non-Hispanic/Latino	350 (74.0%)	102 (86.4%)	248 (69.9%)
Declined to Report	40 (8.5%)	8 (6.8%)	32 (9.0%)
Income level (Federal poverty level %)			
≤100	308 (65.1%)	74 (62.7%)	237 (66.8%)
101–200	58 (12.3%)	14 (11.9%)	43 (12.1%)
201–300	20 (4.2%)	10 (8.5%)	10 (2.8%)
300+	21 (4.4%)	7 (5.9%)	12 (3.4%)
Unknown	66 (14.0%)	13 (11.0%)	53 (14.9%)
Insurance category			
Medicaid	302 (63.8%)	74 (62.7%)	227 (63.9%)
Medicare	3 (0.6%)	2 (1.7%)	1 (0.3%)
None/Uninsured	35 (7.4%)	7 (5.9%)	27 (7.6%)
Other Public Insurance Non-CHIP (incl. DC Alliance)	54 (11.4%)	0 (0.0%)	55 (15.5%)
Private	79 (16.7%)	35 (29.7%)	45 (12.7%)
Parity			
Nulliparous (no prior births)	171 (36.2%)	49 (41.5%)	122 (34.4%)
Multiparous (≥1 prior births)	275 (58.1%)	59 (50.0%)	216 (60.8%)
Grand-multiparous (≥5 previous births)	22 (4.7%)	8 (6.8%)	14 (3.9%)
Unknown	5 (1.1%)	2 (1.7%)	3 (0.8%)
Preferred language			
English	390 (82.5%)	114 (96.6%)	276 (77.7%)
Language other than English	83 (17.5%)	4 (3.4%)	79 (22.3%)

### Effectiveness

In 2022, the COH postpartum visit attendance rate was 78% and increased to 84% in 2023. This increase was at least partially driven by the inclusion of postpartum home care, with data showing substantial increases in visits between postpartum days 2 and 14. In 2023, we performed 873 postpartum visits with 176 (20%) at home. Nearly half (42%) of those who elected in-home care had more than one home visit. In 2023, we conducted a total of 971 newborn visits, 183 (19%) at home. In the first 28 days of life, 132 (28%) infants received at least one home visit. One-third of infants with home visits had additional home visits. The rates of home visits are lower compared with demand; infants often needed to be seen within 24–48 h of discharge and, given limited availability (∼2 days/week), this was not always possible. Similarly, there was higher demand for repeat home visits than there was availability, as we prioritized the early visits (<2 weeks). See Table 4 for details on the distribution of postpartum visits.

**Table 4.** Postpartum visit attendance, timing, and location at Community of Hope

Number of birthing people attending postpartum visits at interval time points Pre and postimplementation of home care		
Postpartum visit timing	Client EDD Jan–Sep 2022 *N* = 244, *N* (%)	Client EDD Jan–Dec 2023 *N* = 473, *N* (%)
<1 week	39 (16%)	183 (39%)
2–14 days	97 (40%)	255 (54%)
7–21 days (2-week visit)^a^	147 (60%)	325 (69%)
22–42 days (5-week visit)^a^	95 (39%)	209 (44%)
22–84 days	166 (68%)	338 (71%)
7–84 days (HEDIS)	190 (78%)	396 (84%)
2–84 days	196 (80%)	401 (85%)

**Postpartum visits trends** **(Estimated due dates Jan 1-Dec 31 2023)**		
	**Birthing people, *N* = 473** **All Languages**	**Birthing people, *N* = 394** **Preferred Language English** ^b^
**Total # *postpartum visits (2–84 days)***	873	730
Patients with at least 1 home visit	118 (28%)	118 (30%)
Patients with 2+ home visits	49 (42%)	49 (42%)
No. of visits completed at home	176 (20%)	176 (24%)
No. of visits in completed clinic	697 (80%)	554 (76%)

**Newborn Visits Trends** **(Estimated Due Dates Jan 1–Dec 31, 2023)**		
	**Newborns, *N* = 478** **Parent all languages**	**Newborns, *N* = 408** **Parent preferred language English** ^b^
***Total # newborn visits (Birth-28 days)***	**971**	**826**
Patients with at least 1 home visit	132 (28%)	123 (33%)
Patients with 2+ home visits	43 (33%)	47 (38%)
No. of visits at completed home	183 (19%)	178 (22%)
No. of visits completed in clinic	788 (81%)	648 (78%)

^a^ Community of Hope standard is to have a 2-week and 5-week postpartum visit, based on Healthy Start and other funding partner recommendations.

^b^ Include four clients whose EHR record noted a preferred language other than English but who were fluent English speakers.

EDDs, estimated due dates

While not a measured outcome, we observed that numerous emerging complications were identified and addressed during early home visits including preeclampsia, mood disturbances, surgical complications, intimate partner violence, lactation problems, excessive neonatal weight loss, and jaundice. Given that early (<2 weeks) postpartum visits are not included in routine postpartum care in the United States, those complications may not have been identified until later visits or exacerbation of the conditions, either of which could be associated with increased morbidity. In some cases, patients were able to avoid emergency room visits by accessing same-day in-home care. This occurred on a small number of occasions when same-day home visits were available and our team deemed the care appropriate for outpatient management.

### Adoption

Among clients who did not have a postpartum visit at home, scheduling was a primary reason. Home visits were not always available in needed time frames, for either routine or urgent health concerns. Initial newborn visits must occur 1–3 days after hospital discharge, thus most newborns seen for in-home care also had at least one in-clinic visit. Parents were sometimes told by hospital staff (unaware that blood work can be drawn at home) that a home visit was not appropriate due to newborn’s blood work. Other clients planned multiple obligations out of the home on the same day, or just wanted to get out of the house. Some clients desired LARCs or were due for a vaccine, both of which are only available in the clinic. A number of clients reported that they like coming into COH and seeing clinic staff.

In-home care successfully addressed some of the barriers and themes from the focus groups of Black birthing people in Washington DC by eliminating the need to arrange transportation and childcare, increasing involvement of older children and support people in visits, providing more thorough lactation support, and having more time for discussion of mental and social well-being and newborn care. Our team noted two broad profiles of patients who tended to opt into home visits.

*Patients who selected COH for its midwifery-led continuity of care model and comprehensive services*. Postpartum home care aligned with their desire for low-intervention care by minimizing disruptions and promoting postpartum rest. Patients who desired a birth center birth but changed to a hospital birth particularly appreciated postpartum home care as a return to a less medicalized model and an opportunity to process grief associated with the loss of birth preferences. Other patients were highly engaged with COH’s comprehensive services including perinatal care coordination, integrated emotional wellness, and prenatal group care. These clients were excited to engage with new home care options.

*Patients with complex medical and/or social needs.* Patients who had limited prenatal care were interested in postpartum home care, as it addressed barriers they faced in accessing prenatal care. Many of these patients expressed a desire for a prenatal home care option. Patients who had birth-related complications or unexpected social circumstances occasionally scheduled clinic visits but changed to postpartum home visits. Health-related reasons for this switch included unexpected surgical birth making it difficult to get to the clinic 3–5 days postpartum, or need for short-term follow-up for blood pressure evaluation, neonatal jaundice, and neonatal weight loss or feeding difficulties. For some patients, we were to conduct home visits within 24 h of discharge. Some patients changed to home care due to social circumstances such as depressed mood, fear of traveling with an infant, and interruptions in social support.

The following video https://youtu.be/g6kW2xK2AH8 follows one of the midwives at COH to a home visit with a mother and baby dyad, highlighting some of the care and joy that is often associated with in-home care. Additionally, the home care midwives generated composite vignettes to illustrate the ways postpartum midwifery in-home care met the needs of patients across the spectrum of surviving to thriving. (See Supplementary Data S1)

### Vignette 1: Navigating social and medical complexity

Jessica, a 32-year-old mother of three, had her first home visit 7 days postpartum. She was experiencing chronic back pain due to a previous injury, which made it difficult to comfortably hold and feed her baby who she described as fussy. Her blood pressure was elevated, and she was tearful about her partner’s recent deployment. She had no reliable transportation. She initially planned for a clinic visit, but switched to home after missing her rideshare because they departed while she tried to get her three children into the waiting car.

Following routine maternal and infant assessments, Jessica and the midwife explored ergonomic feeding positions using household items and seating options to reduce back pain. The midwife demonstrated soothing techniques for the baby’s gas pain and stretches to alleviate Jessica’s back pain. While Jessica took a long awaited shower, the midwife held the baby, consulted with collaborating Ob/Gyns to adjust her blood pressure medication, and sent a prescription to the COH pharmacy for home delivery later that day. Jessica’s brother, her primary support person, arrived during the visit and received teaching from the midwife on how to support Jessica. The midwife initiated a conference call with Jessica’s perinatal care coordinator to arrange rides to future appointments, lactation support, and emotional wellness follow-ups. With the help of a multidisciplinary team, Jessica received holistic care, addressing her physical pain and emotional challenges while supporting her to care for herself and her baby more comfortably.

### Vignette 2: Family-centered joy and connection

Amani, a 29-year-old mother of four, had her first home visit 3 days after giving birth with midwives at our out-of-hospital birth center. She and her newborn daughter were thriving, enjoying rest and bonding as a family. Amani was excited to experience postpartum care at home for the first time. Amani’s three older children and partner eagerly participated in the home visit. The children helped measure their baby sister’s length and weight, and took turns listening to their hearts through the stethoscope. The family took photos and laughed together. The health checks confirmed that Amani and the baby were healthy.

When the midwife returned for the 2-week home visit, the three older children enthusiastically greeted the midwife. They shared pictures they had drawn of their experience helping with the first visit. At that visit, Amani noted that having the whole family present had made it a joyful experience that brought the family closer together, helped the older children settle into having a new baby in the house.

### Vignette 3: Balancing demands and overcoming barriers

Latoya, a 34-year-old single mother of two, called the clinic to schedule a clinic appointment for concerns about her 4-day-old daughter’s jaundice and persistent poor latch. Unfortunately, the night before, she had to take her grandmother to the ER. Her grandmother was discharged home in improved condition at 6 a.m., but Latoya was too exhausted to come to clinic.

When she called to cancel, the care team offered a same-day postpartum home visit. The midwife assessed for signs of jaundice and drew bilirubin levels. The midwife then provided lactation support. Latoya became tearful, sharing that she had stopped breastfeeding her first child sooner than desired because she had no support. She had been very anxious that breastfeeding would not work this time either. Having the home visit option ensured that Latoya’s newborn received a timely jaundice assessment, reduced the cognitive and logistical strain for Latoya caring for herself and her family, and empowered her to continue breastfeeding.

### Implementation

The exact need for home visit availability was not reliably predictable due to fluctuations in weekly births; however, we determined that 2.5 days of availability per week would meet most demand for home visits and was sustainable for overall clinic staffing. During 2023, we had one dedicated home visit midwife, which limited availability during their vacation time and parental leave. Some patients who requested home care were seen in the clinic due to a lack of availability, although we did not track this. Home visit engagement is likely lower than demand due to limited availability during the pilot period.

We made iterative changes during program implementation based on midwife and staff observations and patient feedback. A full visit day included four stops, with up to eight visits if both birth parent and infant were seen. Initially, we scheduled home visits for a specific time; however, extended visit times and varying distances between appointments made precise scheduling difficult. We changed to a 2-h window block scheduling with a confirmation call the day before to confirm address, enabling the midwife to plan their route. In January 2023, we implemented a 30-min driving limit from any COH clinic in order to ensure the availability of four-stops per day. This became important as some clients stayed with family in the immediate postpartum up to an hour outside of DC.

During the confirmation calls, we also assessed for material needs that COH can provide (e.g., breast pumps and supplies, diapers/wipes, pack and plays) and other care coordination needs (e.g., emotional wellness or sexual health coordinators) promoting multidisciplinary coordination.

The midwives who provided in-home postpartum care reported high levels of satisfaction with longer visit times (60–90 min vs. 20 min in clinic) that enabled comprehensive care at a slower pace, increased patient comfort, and closer alignment with the midwifery model of care. Long visits facilitated attunement, storytelling, reflection, family involvement, education, goal setting, and, ultimately, increased perception of trust and mutual respect.

## Discussion

In the United States, structural inequalities create conditions where racialized and minoritized people are more likely to experience inadequate health care access, systemic racism, and stressors, resulting in perinatal health inequities.^31,32^ While efforts focused on survival often aim to prevent severe outcomes by targeting only the most vulnerable populations, these approaches often fail to capture the full spectrum of what families need.^33,34^ Promoting equity requires that we expand this understanding to include joy, connection, and well-being, to support flourishing across communities.

The relaxed pace of postpartum care in the home allowed more time for listening to patients and families, answering questions, and providing health education. Extended visits allowed time for birth processing and storytelling, lactation/feeding support, education about infant care, inclusion of older siblings and support people, discussions about health promotion and management of chronic conditions, and planning for preventive care. Global perinatal health guidelines recognize postpartum home visits by midwives as a critical standard of care with the World Health Organization recommending at least two home visits within the first week postpartum (24–48 h and 3–7 days after birth).^35^ The International Confederation of Midwives emphasizes that these visits are integral to a continuum of care that addresses physical, emotional, and psychological needs.^36^ Yet significant gaps exist across the United States, where many patients encounter fragmented services and inadequate support in the postpartum period.^37^ More than half of pregnancy-related deaths occur in the postpartum period, with about half of those deaths occurring after 6 weeks postpartum, when standard postpartum care ends in the United States.^38^ Overall postpartum visit attendance rates in the United States are only 72%, with a lower rate among those who are Medicaid recipients (62.8%). Due to the baseline inadequate frequency of standard postpartum visits in addition to the low attendance rates at those visits, it is estimated that 40% of birthing people in the United States do not receive adequate postpartum care.^39^

We noted that many birthing people stay with family members during the postpartum period, most citing an increased need for support during this time. However, this often means staying farther from established health care providers, which can create a barrier to follow-up due to longer travel distances, a reliance on public transit, or a need for reliable transportation and childcare. This tendency could be the result of structural challenges, such as a need for parental leave policies for partners or a lack of affordable childcare for other children, which could support families in the postpartum period and potentially reduce these types of tradeoffs that families face.^16^

Extreme weather events disproportionately disrupt health services in structurally disadvantaged communities.^40^ Such events occurred multiple times during our study including extreme heat, snow/ice storms, and a stay-at-home air quality advisory due to wildfires that can pose significant risks, especially for newborns and postoperative patients.^41^ These extreme weather events are a result of rising global temperatures and human-induced climate crisis and are estimated to increase in severity and frequency, highlighting a more urgent need for the expansion of in-home health services to provide safe and equitable care.^42^

In a clinic setting, providers are in a default position of power that is emphasized when patients need to defer to clinic protocols. This power imbalance can act as a barrier to care if it influences comfort and trust.^43^ As providers, we have the privilege of being allowed into vulnerable parts of a patient’s life when they choose to collaborate with us. Providing care in the home shifts power to the patient and can be a means of honoring the responsibility that comes with the privilege of providing care.^44,45^ Additionally, postpartum care models that are rooted in Black feminism emphasize a holistic and person-focused approach to care.^7^ In-home care that shifts power to patients and allows for longer visits and more attention may be an effective approach to providing care within such models.

Implementing in-home care requires thoughtful design and protocols to ensure the well-being of postpartum providers including comfort with driving, access to a reliable vehicle, mobility/ability restrictions, allergies, limited bathroom access, comfort around animals, newborn and lactation care experience, reliable phone/internet, and the potential need for multiple state licenses. Other priorities for our team were: an understanding and practice of antiracism, familiarity with local resources, and comfort with a harm-reduction approach to care. Health care personnel are mandated reporters of child welfare concerns; therefore, in-home care comes with additional vulnerability of families, especially if the clinicians lack an understanding of the historical and ongoing harms associated with carceral systems and the policing of Black families.^46^ Child welfare systems, such as child protective services, are known to disproportionately harm Black families who receive twice as many reports and 2–5 times as many investigations when compared with white families.^47^ Additionally, we believe providers must have proficiency in attunement and de-escalation in order to avoid causing harm. While we encountered no safety concerns during home visits, it is also essential to plan for provider and patient safety in the home setting.^48,49^

To address barriers for postpartum patients, it is essential to evaluate the financial feasibility of solutions that prioritize both accessibility and high-quality care. Medicaid reimbursement for postpartum health care with a focus on thriving could be an essential tool for increasing adoption of this approach by health care organizations and therefore could increase the availability of these services.^50,51^ Staffing models that ensure care accessibility will be necessary to provide comprehensive and equitable care that accounts for the diverse needs of birthing people, helping to address low postpartum visit rates and improve perinatal health outcomes.^16^

## Conclusions and Health Equity Implications

In considering health equity implications and the conditions needed for optimal health, we acknowledge that justified distrust in the health care system is a major barrier and will continue to be if communities are not respected or trusted to lead change. In-home postpartum care may be an effective approach to reducing barriers and increasing visit attendance rates in a health care system afflicted with systemic racism and poor perinatal outcomes. Postpartum care at home can increase family involvement at visits, support longer and more comprehensive visits, and increase provider satisfaction, which may combat high rates of burnout. Most profoundly, in-home care has the potential to increase trust by shifting power to the patient in the patient–provider relationship.

## Positionality Statements

E.C. is White, American, cisgender, heterosexual, able-bodied nurse-midwife. S.C. is an Indian American, cisgender, heterosexual, able-bodied woman. E.E. is a White, American, cisgender, bisexual, able-bodied woman. N.V.J. is a White, Hispanic American, and able-bodied woman. B.M. is a Muslim, Black American woman, nurse-midwife, and lactation consultant. S.S. is a Black, nonbinary, queer, able-bodied, midwife who was raised moving between Senegal, Ethiopia, Haiti, and Ohio. D.S. is an African–American, cisgender, heterosexual, able-bodied, community-driven social scientist, parent, and wife who values diversity in all its forms.

## Abbreviations Used

CHIP: Children's Health Insurance Program
CNM: Certified nurse midwife
COH: Community of Hope
DC: District of Columbia
ECW: EClinicalWorks
EDD: Estimated due dates
EHR: Electronic health record
FQHC: Federally qualified health center
HEDIS: Healthcare Effectiveness Data and Information Set
HIPAA: Health Insurance Portability and Accountability Act
IEW: Integrated emotional wellness
IRB: Institutional review board
LARC: Long acting reversible contraceptive
MCH: Maternal and child health
MD: Maryland
Ob/Gyns: Obstetrician/gynecologist
PCC: Perinatal care coordinator
QI: Quality improvement
RE-AIM: Reach, Effectiveness, Adoption, Implementation, and Maintenance
WIC: Supplemental nutrition for women, infants, and children
